# Medical Association of Georgia

**Published:** 1860

**Authors:** A. G. Thomas

**Affiliations:** Sec. of the M. A. of Georgia


### Medical Association of Georgia.

The annual assemblage of the Medical Association of Georgia, for 1860, will take place in the city of Rome, on the *Second Wednesday* in April next. Will the Medical and Newspaper press of the State, extend this notice to their readers. A. G. THOMAS, M. D.,

Sec. of the M. A. of Georgia.

